# ‘Mind the gaps’: stakeholder perspectives on addressing antimicrobial resistance in the environment in the Indian context

**DOI:** 10.1080/16549716.2025.2491200

**Published:** 2025-05-01

**Authors:** Anishka Cameron, John Connolly, Regina Esiovwa, Fiona L. Henriquez, Andrew Hursthouse, Suparna Mukherji, Soumyo Mukherji

**Affiliations:** a School of Health and Life Sciences, University of the West of Scotland, South Lanarkshire, Paisley, UK; b Glasgow School for Business and Society, Glasgow Caledonian University, Glasgow, UK; c Scottish Antimicrobial Prescribing Group, Healthcare Improvement Scotland, Glasgow, UK; d School of Computing, Engineering and Physical Sciences, University of the West of Scotland, Paisley, UK; e Environmental Science and Engineering Department (ESED), Indian Institute of Technology Bombay, Mumbai, India; f Department of Biosciences and Bioengineering, Indian Institute of Technology Bombay, Mumbai, India

**Keywords:** Stakeholder engagement, one health, manufacturing waste, pharmaceutical pollution, policy, qualitative study

## Abstract

**Background:**

There is growing global awareness of the pivotal role environmental factors, including pharmaceutical manufacturing waste, play in the development and spread of antimicrobial resistance (AMR). India bears one of the highest burdens of AMR globally and possesses a substantial manufacturing sector, but limited insight is available on how to practically mitigate environmental AMR-related risk in this context.

**Objective:**

To understand the barriers and opportunities in managing manufacturing waste for addressing AMR in the environment from the perspectives of stakeholders in India.

**Methods:**

We conducted semi-structured interviews with a range of stakeholders from government, industry, and civil society following a stakeholder mapping and analysis process within the Indian context. We also undertook a series of stakeholder events to inform the study.

**Results:**

Our findings indicate that 1) Policy action is fragmentary and there are economic and capacity gaps that have implications for industry behaviours; 2) A One Health approach to addressing AMR in the environment requires leadership and that means AMR prevention needs to be institutionalised within government for them to steer, facilitate and coordinate; and 3) There is a need to enhance knowledge amongst policymakers in India about AMR in the environment, and robust ‘evidence’ is required to foster policy change.

**Conclusions:**

The study underscores the need for a multifaceted strategy to address the contribution of pharmaceutical manufacturing waste to AMR in the environment in India. Greater prioritisation of AMR, stakeholder collaboration, and capacity building are essential to overcoming the challenges identified.

## Paper context

- **Main findings:** Capacity constraints impede the implementation of regulatory frameworks in this area, and it is necessary to balance the demands of reducing AMR with pragmatic solutions that consider economic realities and infrastructural limitations.
- **Added knowledge:** The study provides new insights into stakeholder perspectives and strategies to address the contribution of pharmaceutical waste to AMR in the environment in a lower-middle-income country.
- **Global health impact for policy and action:** The findings emphasise the critical need to integrate AMR prevention within governmental structures and highlight the importance of understanding country-specific contexts to support effective and sustainable alignment with future international standards on managing manufacturing waste.

## Background

In tackling the formidable challenge of antimicrobial resistance (AMR), this study stands at the forefront of international research efforts by examining the crucial intersection of environmental factors and pharmaceutical manufacturing, with a focus on India - a country central to global health dynamics. AMR represents one of the most pressing global health challenges in modern times, posing serious risks to public health, food security, and economic stability [1]. In September 2024, world leaders endorsed a political declaration on AMR, reaffirming its significance on the global agenda and highlighting an urgent need for priority actions to address the environmental factors contributing to it [2]. This reflects the growing awareness in recent years of the critical role the environment plays in the development and spread of resistance [3–5]. Indeed, the environment is a fundamental component of the multisectoral One Health approach that underpins many national action plans (NAPs) to address AMR [6,7].

The environment serves as a reservoir of antibiotic-resistant bacteria (ARB) and antimicrobial resistance genes (ARGs) that pose exposure risks to humans and animals through pathways including food and water consumption, as well as direct contact [4,8]. Environmental ARB, where resistance can naturally occur, can act as sources of ARGs with the potential for horizontal gene transfer (HGT) to human- and animal-associated pathogens [4,9]. Conversely, ARGs from human- and animal-associated pathogens may also transfer to environmental bacteria [10], where they can persist and be harboured. These ARGs can later re-enter such populations, continuing the processes of resistance.

Although knowledge of the dynamics and extent of transmission is still developing [11,12], research in United Kingdom (UK) coastal waters have found that surfers are at an increased risk of colonisation by resistant *Escherichia coli (E. coli)* [13,14]. Human exposure to resistant *E. coli* through the consumption of lettuce irrigated with surface water has also been modelled [15]. Moreover, even rare HGT events are capable of triggering widespread and significant impacts [8,9]. Environmental monitoring and surveillance are therefore critical components in guiding AMR initiatives, identifying sources, exposure routes, and risk, as well as informing trends and changes in resistance patterns [16,17].

The potential for anthropogenic activities to exacerbate the risk of AMR in the environment is a significant concern. Pollution contaminated with antibiotics (even at low levels) can exert selective pressures, promoting the proliferation of resistant microorganisms, encouraging mutations, and facilitating HGT [18,19]. The elimination of antibiotic residues, ARB, and ARGs is a substantial challenge that conventional wastewater treatment systems are not equipped to address, inevitably leading to their release into surrounding environments [20–22].

Several sources can contribute to antibiotic pollution (e.g. human and livestock waste) [5,23]; however, research frequently associates prominent levels with pharmaceutical manufacturing waste [24–28]. For example, a study in India revealed that a common effluent treatment plant (CETP), which processed waste from around 90 pharmaceutical manufacturers, discharged up to 45 kg of ciprofloxacin per day [26]. Yet, there are no regulations that address antibiotic discharges from pharmaceutical manufacturing [29]. Although there is guidance around discharge limits available from the AMR Industry Alliance (AMRIA) and, more recently, the World Health Organization (WHO), implementation and widespread adoption remain a significant challenge [30,31]. Policy-focused research is therefore crucial for understanding these challenges and supporting the development of effective strategies.

This study examines the Indian policy context of AMR in the environment by exploring stakeholders’ perspectives, focusing on pharmaceutical manufacturing waste management. India is a lower-middle-income country (LMIC) facing one of the highest rates of AMR burden [32] and is at ‘severely high risk of becoming the AMR capital of the world’ [33]. Several studies in India have identified very high concentrations of antibiotics within manufacturing effluents. These effluents have been associated with the pollution of surrounding rivers, as well as surface, ground, and drinking water, and increases in ARB and ARGs [26,34–37]. Recognising this pressing concern, India stands as one of the few countries that addresses the need for action within its AMR National Action Plan (NAP) [38]. By studying India’s approach, valuable insights can be gained in managing this challenge in an LMIC context, which can also help to inform AMR strategies globally.

## Policy context of India

Although the Indian NAP has been outdated for several years now, the 2017–2021 cycle aligned with a One Health approach and contains several strategic priorities that incorporate the environment [39]. Pharmaceutical manufacturing waste was addressed under strategic priorities 2 and 3, which emphasised the development of a national surveillance framework for antibiotic residues, including those from the pharmaceutical industry, and the definition of standards for these residues in effluent [39].

The Indian pharmaceutical industry is the leading provider of generic drugs globally and the third largest producer, including the manufacture of antibiotics [40,41]. The country exports substantially more drugs than it imports, with the pharmaceutical sector contributing to around 1.72% of India’s GDP [40]. There are more than 3000 pharmaceutical companies across India, including over 10,500 manufacturing facilities [42]. Antibiotic manufacturing is primarily distributed across 25 hubs, which span nine different states [43].

Guidance on pharmaceutical effluent standards in India is provided by the Central Pollution Control Board (CPCB), under the Ministry of Environment, Forest and Climate Change (MoEFCC), which coordinates with each State Pollution Control Board (SPCB) to ensure compliance with environmental laws and regulations [44]. However, antibiotic residues are not currently monitored within these frameworks [45].

The CPCB mandates pharmaceutical companies of all scale to adopt a Zero Liquid Discharge (ZLD) scheme [46], which recovers water for purification and reuse while concentrating solids for disposal. Although many bulk drug manufacturers have adopted ZLD systems, often in conjunction with other practices, technologies specifically for managing antibiotic residues are rarely applied [46]. Moreover, monitoring for ZLD compliance is lacking, and not all pharmaceutical companies claiming to operate ZLD adhere to the scheme appropriately [47].

In early 2020, the MoEFCC published a draft ‘Environment (Protection) Amendment Rules, 2019’, proposing legally binding limits for 121 antibiotics in pharmaceutical manufacturing effluent. This placed India at the fore of action against pollution from pharmaceutical companies globally, as the first state regulator to introduce antibiotic discharge standards [48]. However, the final notification, released in mid-2021, reflected a complete removal of antibiotic limits, and the rationale behind this decision remains unclear [49,50]. Efforts to implement the original draft limits in India are, nevertheless, ongoing, with stakeholders and judicial bodies such as the Veterans Forum for Transparency in Public Life (VFT) and the National Green Tribunal (NGT) actively engaged [43].

Overall, the policy landscape related to the issue of manufacturing waste for AMR in India is multifaceted, marked by a substantial manufacturing presence, and involving a variety of stakeholders and varying levels of regulatory enforcement. A comprehensive understanding of these factors is therefore crucial for effectively mitigating the impacts of manufacturing waste on AMR in the environment and advancing India’s One Health approach.

## Methods

### Study design

This study employed a qualitative approach to investigate the perspectives of stakeholders involved in AMR in the environment and antibiotic manufacturing waste management in India. Our theoretical assumption is that the effectiveness of stakeholder engagement in addressing AMR is influenced by the relative power and interests of stakeholders. The approach was based on stakeholder theory, which recognises the complexities within policy systems and that mapping the interests and power dynamics of stakeholders need to be understood to make sense of the landscape (see for example [51–53]). In this study, stakeholder power refers to the ability of a stakeholder group to influence decision-making processes, while stakeholder interests refer to the specific goals or concerns that a group seeks to satisfy. The interplay between power and interests is crucial, as it reveals how different groups shape policy decisions on AMR in the environment. This is because, as outlined in our background section, the management of antibiotic waste is a complex issue, requiring coordination and cooperation among multiple stakeholders.

In the first instance, an in-person stakeholder event was organised at the Indian Institute of Technology Bombay (IIT-B). This was done to facilitate stakeholder engagement and to understand stakeholder perspectives regarding AMR associated with antibiotic manufacturing waste. The event was held in January 2023 and stakeholders with interests in AMR in India were invited. As part of the event, a focus group discussion was held. This investigated the challenges and opportunities within antibiotic manufacturing wastewater management. Attendees were representatives from research/education, health, manufacturing, and the third sector. Minutes were taken and subsequently reviewed. This was done to gain valuable insights and to inform the interview questions in the next research stage.

**Figure 1.**
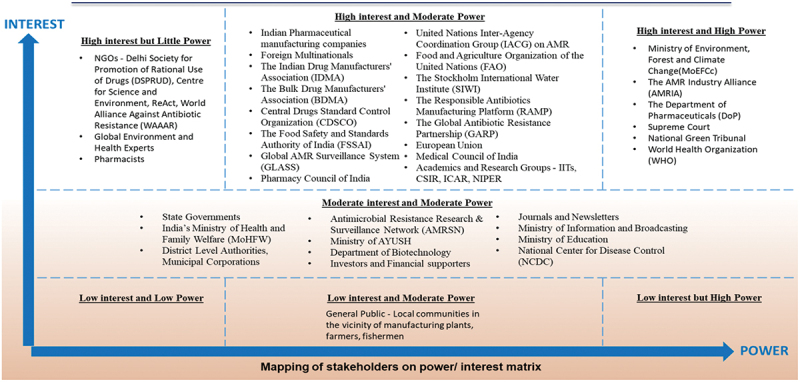
Stakeholder interest grid: AMR in India.Source: Yadav [54] with contributions from JC, RE, SM, and SM.

Secondly, grounded in desk-based research and the focus group discussion, a stakeholder mapping exercise was conducted to ensure that all relevant stakeholder perspectives were captured (see Figure 1).

The exercise included a stakeholder identification process followed by the creation of an interest – influence matrix.

- Stakeholder identification: A desk-based literature search was conducted on PubMed to identify publications on antimicrobial manufacturing and its impact on AMR. Articles were included if they conducted research on pharmaceutical wastewater analysis or management, highlighted pollution concerns, or proposed solutions to address this issue. Grey literature was searched for relevant publications, as were newspaper articles in India. Author name and affiliation were extracted from the publications and collated on a data collection form. Knowledge of author affiliation facilitated stakeholder categorisation into sectors, i.e. government, manufacturing, third sector, and education/research.
- Stakeholder interest and influence matrix: An online survey was created using Google forms. The survey included the list of identified stakeholders, and the survey responders were asked to rate the stakeholders as low, medium, or high for influence and interest based on the responders’ own views. Opportunistic sampling of the stakeholder list was used to identify the responders. Responses were collated, and stakeholders were grouped from low to high for influence and interest. The information was subsequently inputted into a matrix for visual presentation.

### Data collection

Stakeholder interviews were conducted on the basis of focusing on stakeholders identified as high influence or high interest. An email requesting an interview was sent to these stakeholders and responders who agreed to be contacted were subsequently interviewed. Interviews were semi-structured and included questions that would facilitate the identification of challenges, opportunities, and practical solutions to antibiotic manufacturing wastewater management. Interviews were held online and transcribed from video to word. All interviews were conducted between March and May 2023. In total,12 interview meetings involving 15 participants were conducted including six non-governmental organisations (NGOs), three manufacturing organisations, two research institutions, and one policy/regulatory organisation.

### Data analysis

This exploratory research sought to understand emergent themes based on stakeholder perspectives. In this respect, a thematic analysis approach [55] was applied to identify common themes, nuances, and complexities within the interview data. Coding of the data was carried out independently by JC, RE, and AC using NVivo. The key themes were agreed between the researchers in the interests of within project cross-validation. The interview quotes were then grouped into key themes, and these are represented in the headings that are to follow in the findings for this paper.

An additional in-person stakeholder event at IIT-B was held in December 2023 at which preliminary findings were shared, and stakeholders were invited to discuss these further. This was done to validate the interview findings with a broader group of stakeholders and stay up to date with the challenges and opportunities in India. Attendees were representatives in research/education, health, pollution control, manufacturing, and the third sectors.

### Ethics

The research was conducted in accordance with the University of the West of Scotland’s Code of Ethics and Guidelines for Ethical Practice in Research and Scholarship. All participants were provided with information on the aim and nature of the research and gave informed consent to participate in the study. The data collected were pseudo-anonymised and stored securely.

## Results

Four main themes were categorised from the interview data, including several subthemes. An overview is presented in Table 1.

**Table 1.** Themes and subthemes of stakeholder perceptions.

Theme	Subthemes	Description
Importance of evidence	Comprehensive evidence-informed frameworks	Discusses the need for methodological standards, and data that is source-specific and credible
	Scientific uncertainties	Discusses challenges associated with limit values and the need to balance precaution with practicality
Navigating policy complexities	Economic and capacity constraints	Discusses challenges related to resource, infrastructure, and expertise
	Balancing the risk of unintended consequences	Discusses the potential implications of discharge limits on the wider antibiotic supply chain
	Policy conflicts	Discusses the tensions between government and industry priorities and approaches
Turning policy into action	Enforcement	Discusses governmental enforcement challenges
	The role of incentives	Discusses alternatives to regulation
Bridging perspectives for the future of AMR in the environment	AMR is a One Health issue	Discusses the variety of sources that influence AMR in the environment and the need for collaboration
	Awareness	Discusses the need for broader stakeholder AMR awareness and associated challenges
	Leadership	Discusses the role of government and international organisations in addressing AMR in the environment

### Importance of evidence

Evidence emerged as a key point of discussion across the interviews, highlighting its fundamental role in shaping understanding, influencing policy decisions, and promoting action. Different data needs and dynamics were identified that impact balanced and collective progress to address AMR in the environment.

#### Comprehensive evidence-informed frameworks

Stakeholders posed several questions highlighting a lack of understanding and agreement on best practice monitoring and surveillance methods, including *how* (the methods) to sample and detect antibiotic residues and AMR in wastewater, *what* to monitor (priority antibiotics, ARGs, ARB), and *where* to monitor (point of discharge/receiving environment):

First of all, what are the suitable methods to detect AMR in wastewater? This needs to be defined first. If define the methods, then the parameters applied are also important. (Manufacturing3)

Data connecting antibiotic concentrations in the environment to specific sources was viewed as essential by several stakeholders. This data represents a ‘first step’ (Policy1) supporting change, by facilitating the pinpointing of responsibilities:

I think there is definite need for generating more data, like first you have to generate the data, how much is coming from where? Which one is responsible for which one. And not all companies maybe contributing equally to one. Of course the one’s that produce API will be the highest ones. (NGO6)

However, the impact of AMR-related environmental data appeared influenced by credibility perceptions. This was primarily informed by data ownership, with pharmaceutical companies facing lower levels of trust than government-led institutions:

I’m talking about subject neutral matter of policy making … how credible the data is in your hand and who has owned that data. Whether any Government Institution has owned that data. Like suppose if I come out with some data from some private firm where they will not accept that data. (Research1)

So if you have spoken with the [pharmaceutical association] of India, they will keep referring to a study which they sponsored, and obviously the data is consequently in question. (NGO6)

#### Scientific uncertainties

The risk of antibiotics in the environment to human health was emphasised as a complex and evolving area, impacting our ability to effectively assess impacts.

So right now it’s our understanding that the weight of science is such that you can’t assess the human health risk from the concentration of an antibiotic in the environment. You just can’t do it … That’s the challenge that we face. (Manufacturing 1)

This poses significant difficulty in meeting the data demands suggested by some stakeholders as necessary to gain political attention and secure investment:

You see, the Environment Ministry and particularly the Health Ministry in all the states, they ask the same questions. ‘Show us the data that, you know, the environment is suffering specifically because of what’s happening in the factories.’ And the Health Department says, ‘Show us one dead person because of what’s happening in the factories. And when you do, we will be the first to stop all this’. (NGO5)

The above quote additionally contains overlap with the One Health theme (discussed later), as it distinguishes the environment as a separate dimension with its own health status. Though the environment is typically perceived as a medium that influences the health of humans and animals, it can be recognised that the risk of antibiotics in the environment are two-fold. Risk assessment therefore encompasses distinct considerations that need to be addressed, which the AMRIA and WHO both recognise in their frameworks for manufacturing waste:

There’s two risks here – there’s the risk to the actual environment from the antibiotic, and of course there’s the risk in terms of AMR, and of course it has a very strong public health element as well. (Manufacturing2)

Predicted No-Effect Concentrations (PNECs) are estimates used in environmental risk assessments to determine the concentration of a substance below which it is expected that no adverse effects will occur in the natural environment.

Key challenges related to the robustness of PNEC values used for antibiotic discharge limits, particularly those proposed by the Indian Government, as well as the appropriateness of PNECs for addressing AMR in terms of the ability to account for the complexities of resistance:

I would not say their [PNEC] numbers were scientific – they were numbers. You know, so we said we do support the conception of regulation in principle. We didn’t agree with the actual numbers they had chosen. (Manufacturing1)

The original PNEC values for resistance, and I kind of hesitate to say this – but they’re not true resistance values. They’re not based on resistance end points. (Manufacturing2)

This ongoing debate around discharge limits hinders a comprehensive and collective approach to addressing AMR in the environment. An interviewee emphasised the need to take action, even if the steps taken are not fully aligned with the precautionary principle, highlighting the importance of balancing precaution with practicality to enable progress:

I think the views on the precautionary principle, for example, are if we argue about that, you could never get past square one on that. And so we’d need to pick a starting point, and we think we’ve picked a viable one that’s based on the science that we have today. (Manufacturing2)

### Navigating policy complexities

A multitude of factors were identified to influence the design and implementation of AMR in the environment policy, emphasising the complexity and tensions associated with the pharmaceutical manufacturing landscape in India and globally.

#### Economic and capacity constraints

In India, prioritisation of AMR, including its environmental dimension, was suggested to be limited. It was explained that health service directors face constraints due to the lack of resource dedicated to AMR, as more ‘visible’ issues tend to command greater policy attention:

You talk to any director of health services in a state, and then tell him that AMR… his first response is, ‘I have no time for AMR. Right from morning until evening I’m just busy with doing ABCDE,’ or all those existing problems which are visible, and for which plenty of money comes from Government, and then he has to utilise that money. For AMR there has not been any dedicated funding. (Policy1)

In terms of addressing the potential impact of pharmaceutical manufacturing waste on AMR, concerns were raised regarding the timeframe and scope of discharge limits. It was stressed that a ‘*stepwise, non-blanket approach’* (NGO4) to setting limits is necessary, enabling gradual industry compliance:

There should not be immediate enforcement from the government authorities. The government also needs to give industry time to develop capability and develop resources in order for the pharmaceutical industry to comply. (Manufacturing3)

This is because, as indicated in the above quote, the implementation of limits will necessitate operational changes and capital investment, for which many pharmaceutical companies lack the necessary resource, staff expertise, or infrastructure to effectively support. It was highlighted that even large-scale companies in India struggle with limited laboratory capacities and often need to send their samples abroad:

And then the capacity building needs. So for example, what we’re hearing is that even the big [company], for example, they are sending their samples from India to Europe to get them tested and analysed. I mean, that’s ridiculous, right? The cost, time, and energy. (NGO5)

However, lower-cost options to reduce pharmaceutical discharges were recognised by industry:

Where are you generating waste, and can you prevent that? Or can you reduce it, for example, by dry cleaning equipment rather than wet cleaning of equipment, or by containing equipment washings and then, say, incinerating those, and not letting those go down the drain? Things like that, they’re not massively expensive. (Manufacturing1)

#### Balancing the risk of unintended consequences

The antibiotic supply chain was emphasised as fragile and susceptible to disruption. For example, increases in production costs can *‘affect the cost of goods’* (Manufacturing 3), impacting medicine affordability. Furthermore, companies might struggle to sustain increased costs, risking market withdrawal and reducing the availability of antibiotics:

I worry the most that overly proscribed policy solutions, or poorly thought-out policy solutions adopted only in certain jurisdictions, could have an unintended effect on disrupting an industry that is already fragile, and that is already running on very, very small margins, financially. And this can impact availability of much needed antibiotics, and ultimately will have a negative impact on patients who need these products. And so that’s our biggest concern in the end, is the unintended consequences down the line. (Manufacturing2)

Proposed solutions must carefully deliberate prospective risks to minimise impacts of unintended consequences. Economic modelling analysis, previously conducted by the Wellcome Trust, was highlighted as a potentially supportive method:

It might be worth your while referring to that work, at least looking at it. So they looked at two different scenarios in this work. One was basically the proposed Indian regulations … and what they did is, they did some economic modelling, looking at the costs that would be incurred in implementing, you know, one and then the other of these approaches. And they looked at the impact on the supply chain. (Manufacturing1)

#### Policy conflicts

Political tensions were identified as a substantial challenge for policy progression in this area, with the political landscape in India suggested to shape prioritisation decisions:

The other challenge is, because of the whole political economy of this sensitive issue, and in India, the Government’s sort of determination to keep India happy, particularly because… industry happy, sorry, because elections are coming and so forth. (NGO5)

Yet, many stakeholders, including industry representatives, appeared supportive of the protective aim of discharge limits. Rather, disagreements centred on the policy approach and role of the Indian Government, which was perceived as non-participatory, overfocused on regulation, and lacking a transparent evidence base:

They took the AMR Alliance’s public limits, and they used some sort of dividing factor to make them even more, shall we say, constrained or stringent. Now, there may be justification for that, but I’m not aware of any justification that was brought forward. And we were not made aware of any… in terms of transparency, how those calculations were actually made. So we found it very curious. (Manufacturing2)

The government agencies are working on policing rather than giving technical guidance to industry. They need to provide technical guidance to the industry … Government should not act as enforcing bodies, they should not involve their ideas. (Manufacturing3)

Industry stakeholders advocated an enhanced participatory approach to policymaking in this area:

This is a technical matter and it’s a serious one for the environment. It requires collaborative work between government and industry. That’s what I feel. (Manufacturing3)

### Turning policy into action

Enforcement of discharge limits was recognised as a considerable challenge in India and many stakeholders emphasised alternatives to regulation as a key pathway to progression for tackling AMR in the environment associated with manufacturing waste.

#### Enforcement

As highlighted by an international policy expert, for example, the impact of policies and regulations depends on enforcement:

It’s very easy to pass a bill and then put it on your website, but then who’s going to enforce it? (Policy1)

Capacities were emphasised as key in enforcing regulations, which the Indian Government were indicated to be constrained by:

This is a problem in India. They, you know, it’s either, it’s all or nothing, and when it’s all, they’ve no way of enforcing it, because they don’t have the resources and the skills or sometimes the technology to even enforce some of this stuff. (Manufacturing1)

#### The role of incentives

Several stakeholders proposed incentivising industry as an alternative to regulation:

Unless we are incentivising the manufacturing units they will not come forward. (NGO1)

I mean, laws is one thing, but I think globally speaking we would be fastest at having incentives (NGO3)

Suggestions of incentives included governmental funding to support production costs and capacity building, monetary rewards, tax breaks for meeting targets, and governmental investments in academic collaborations to provide expertise and mentorship:

I think the government has to develop capability, provide resources to detect the parameters, and also the other systems that are available i.e. hard and soft systems. (Manufacturing3)

So if there are incentives that are given to meet certain targets, and meet them within a stipulated time, right? Or if you, or if one were to say, like, a hundred ppm [parts per million] is my target for a certain antibiotic, and if you reach that target, if you have reached that target in five years, but if you do it in two years I’ll give you a tax break or something like that. (NGO4)

Procurement-based tactics emerged as a promising strategy. Some stakeholders highlighted the role and keen interest of initiatives based in Northern Europe, such as the Stockholm International Water Institute (SIWI) in Sweden, which promotes green procurement:

If they’re not doing this, they need incentives. State Government has to have carrots and sticks, to, you know, sort of A, encourage them to take action, and B, penalise them to not take action. And that’s where also the global procurement landscape comes, and you know, I think a lot of, when it is UK, you know, the Swedes [Sweden] or whatever. They need to put pressure through the procurement work stream. (NGO5)

However, as noted by another interviewee, procurement pressures still carry market risks:

They receive more points, so they can win the tender …, we do not have the alternate because other manufacturers were just withdrawn from the market, because it’s too small for them to stay in the market and to pay the price. (NGO2)

### Bridging perspectives for the future of AMR in the environment

Stakeholders emphasised that a variety of sources can contribute to antibiotic pollution, advocating for a comprehensive (One Health) approach across sectors for which AMR awareness and leadership are essential.

#### AMR is a one health issue

Several interviewees acknowledged the significance of manufacturing waste but emphasised that targeting industry alone is insufficient:

You could solve the manufacturing challenge and will still have concerns about antibiotics in the environment, because they get there from many, many other sources, as doubtless you know. (Manufacturing1)

Also don’t target only industry. Industry manufacturing is a source of pollution, but there are many other sources of contaminations. They include hospitals who are purchasers, medical shops who give us drugs, some agencies that dispose of … It might be going for landfilling also. (Manufacturing3)

The cross-cutting nature of AMR was highlighted across interviews, emphasising the need for a ‘One Health approach … follow[ing] its multidisciplinary principles’ (NGO2). Collaboration among human, animal, and environmental health fields among government, academia, and industry emerged as essential:

And different kinds of representation. Not necessarily only the one or two sectors, but you know, somebody from the research side, somebody from the non-research side, somebody from the operational side. So different kinds of people from the pharmaceutical sector as representatives, and they sit along with academia, hospitals, medical institutions, the policymakers and government officials, so they all have to first sit together to understand this is a problem. (Research2)

#### Awareness

Limited awareness and understanding of AMR emerged as a substantial barrier to collective progress. An interviewee from an organisation that ‘try to advocate One Health approach’ attributed this situation to the technical characterisation and human health-focused narrative that persists around AMR, hindering wider attention:

The healthcare of course they know, but if you go to other non-healthcare departments and you start talking about AMR, not many people understand. So it’s a too, too much technical term, the narrative is too, still restricted to human health side, so there is a need for changing the narrative to a much more broader one, like keeping it as a development issue, or as goal issue or public issue. (NGO6)

Interviewees emphasised the importance of communication in raising awareness at all levels, highlighting the need for input from social scientists to inform strategies:

I can tell you with guarantee that whatever materials is put on the walls, or given to people, the value becomes zero. No-one reads them, and those who read them, they don’t remember it. What we need, and what I have been telling to people even in the health sector, and animal sector, hire good social scientists. Hire good communication experts who can first understand why people are doing it like this. And I’m not talking about only industry. I’m talking of across the board, whether it’s the animal owner or it’s the human being. (Policy1)

#### Leadership

Governments were perceived as drivers of change for tackling AMR in the environment, underscoring the importance of strong leadership. For example, interviewees described the government as having a central role in *‘incentivising production so that they [companies] can then handle better their waste’* (NGO6), getting ‘*on board with*’ the AMRIA certification scheme and *‘start enforcing it’* (Manufacturing2), and facilitating *‘extensive consultations with all the stakeholders’* (Policy1).

Specifically, governments were emphasised to hold ultimate responsibility for determining and implementing action when provided with research evidence. This process was conveyed to involve complex dynamics, with the quotes below contrasting more subtle guidance and assertive instruction, though each relying on governmental authority:

We kind of give our evidence to the Government, and it’s the Government which then decides next step what do they want to do and how do they want to do it. So that’s at the Government level that these decisions are taken, of interweaving. (Research2)

Let’s agree on those principles. Second stage will be to convert them into general guidelines. Let’s develop those guidelines, right? And third will be, then tell the Government, ‘Okay, these are the guidelines for you to implement and develop a policy on monitoring antibiotic residues’. And let the Government go through the process of education awareness to understand the process, bring in the stakeholders’ buy in, and implement a law that says that you have the guidelines to monitor. (NGO5)

However, in India, leadership gaps in addressing AMR, including its environmental dimension, were identified. These are anticipated to persist through the NAP revision:

There is no ownership of the national action plan. And now we are in the process of developing the national action plan 2.0, which will start this year and go up to next five years. We are facing exactly the same problem even now from the environment sector. So, that is one area where, I don’t know who has to take the lead, but some kind of efforts need to be made. (Policy1)

While international organisations were specified to hold an instrumental advocacy role in this area, it was stressed that ultimate responsibility for NAP implementation rests with national governments:

First would be the advocacy for policy, with the environment people. Someone should make people understand and this role should go to the big international development partners … a lot of capacity building activities are taking place across the world, and the WHO is supporting those things or taking the lead… But ultimately, the implementation part of any action plan, especially with the prefix of ‘national’ is the responsibility of the national government (Policy1)

## Discussion

This study advances the global understanding of policy and practice challenges in India relating to addressing AMR in the environment in the context of pharmaceutical manufacturing waste. By leveraging the perspectives of key stakeholders, the findings provide practical insights that can inform and enhance future AMR mitigation efforts both within India and internationally. Consequently, this research adds to the growing body of literature around the development and implementation of environmental monitoring and surveillance activities for AMR [16,56,57]. Overall, this research indicates that industry stakeholders hold a cautious openness to the prospect of antibiotic discharge limits in manufacturing effluent, with a preference for incentives over regulation. Several critical barriers emerged as hindering progress, underscoring the complexity of the policy landscape and a need for stronger leadership to sufficiently address AMR and its environmental dimension in India.

Economic challenges and risks were identified as significant factors contributing to policy resistance and inaction. For example, resource and infrastructural related constraints were emphasised as a major barrier to implementing discharge limits, particularly in the Indian context, which is consistent with the findings of Kotwani et al. and Peters et al. [56,57]. Industry concerns were frequently expressed in terms of the potential for enforcement to translate serious and far-reaching societal implications concerning the affordability and availability of antibiotics. Subsequently, some industry interviewees advocated for incentives, such as government financial assistance, similar to the findings of Nijsingh et al. and Glover et al. [58,59]. Support for incentives (interference) while opposing regulation (non-interference) is a recognised paradox within the literature, which has been suggested to potentially serve as a mechanism to protect industry interests [59].

Incentives as an alternative to regulation were also proposed by several NGO stakeholders. These were perceived as key for getting industry on board with discharge limits, representing the quickest approach to influence global change. Notably, incentives relating to procurement were emphasised as an emerging strategy of value by both NGOs and industry stakeholders. Despite the potential for both negative and positive pressures, procurement-based tactics were typically preferred over regulation. This may be due to incentives generally offering greater flexibility and alignment with the market-driven dynamics of industry.

The AMRIA and WHO frameworks, as mentioned in the background section of this paper, represent key opportunities to create industry procurement pressures by setting manufacturing expectations. An example of this can be seen in the UK, where AMRIA certification has been adopted as a requirement for manufacturers applying to the National Health Service (NHS) antimicrobial product subscription model [60]. However, this approach has been designed to stimulate antibiotic research and development and may not provide the same level of incentive for the existing antibiotics market. As highlighted by the findings, market dynamics are complex, and procurement schemes are not without challenges. In short, careful deliberation and strategic planning are essential to ensuring incentives are effective and serve their intended purpose [58].

The feasibility of implementing incentives or regulation is hindered by uncertainties and the absence of accredited standards [61]. This affects understanding and clarity for manufacturers, creating barriers to the development of consistent and coordinated approaches. Stakeholders emphasised a need for comprehensive frameworks and more tangible evidence to encourage both industry and government prioritisation. In particular, advocating for clear methods and discharge limits driven by credible evidence, alongside data that advances understanding of the risk of pharmaceutical waste on human and environmental health and connects antibiotic pollution in the environment to identifiable sources. The dynamics of AMR in the environment are intricate, and increasing global understanding is crucial for guiding effective action. As stressed by Nijsingh et al., there is sufficient consensus on the associated risks to prompt activity [58].

Governance literature recognises that complexity and uncertainties underpin many contemporary policy issues, particularly those related to environmental health [62,63]. Dewulf and Biesbroek propose that differentiating between the nature and object of uncertainty can support strategies to address them. In the context of the current findings, several types of uncertainty can be identified, such as ambiguity (e.g. varied stakeholder perspectives on solutions), substantive epistemic deficiencies (e.g. gaps in knowledge on the impact of pharmaceutical waste) and substantive ontological (e.g. the unpredictability of environmental interactions). Suggested strategies for navigating such uncertainties therefore include establishing opportunities for learning and negotiation, increasing knowledge sharing and coordination, and building adaptive capacities [62]. Consequently, collaborative forms of governance are advocated as an effective tool for bringing together diverse stakeholders to manage uncertainties in environmental challenges [64].

The findings ultimately underscore a critical need for stronger AMR leadership and the institutionalisation of One Health principles in India. The lack of ownership of the AMR NAP and governmental approach to establishing discharge limits, characterised by limited stakeholder engagement, emerged as major barriers. Several interviewees called for a collaborative approach to addressing AMR in the environment, involving not only industry and government but also academia, health care, and agriculture. This aligns with a One Health approach, emphasising the importance of stakeholder input to ensure that policies are feasible and sustainable. Yet, it must also be acknowledged that some stakeholders might embrace One Health as a means of deflection by emphasising the contribution of other sources. It is therefore crucial to ensure that the interconnectedness between sectors underpinning One Health is understood as promoting active participation and responsibility among all stakeholders.

Finally, it is important to acknowledge the limitations of this study. Despite conducting a comprehensive stakeholder interest-power mapping exercise to inform our approach, the Indian government authorities were reluctant to engage. It is recognised that while the research was part of a North–South collaboration, perceived power dynamics can still play a significant role in stakeholder engagement [65,66]. Furthermore, although communication, interest alignment, and early involvement of policymakers in the research process are recommended strategies, political willingness and absorptive administration remain critical factors [67,68]. The reluctance to engage is a finding in and of itself, but it would have been helpful to gain official government insights. That said, our sample represents a diversity of key voices from important sectors. For the future, we would be keen to expand the geographical coverage of the study, but we were fortunate to be able to engage with stakeholders who have broad and global interests in AMR as well as specific knowledge of the Indian policy environment.

## Conclusion

This study underscores the complex interplay of stakeholder power and interests in addressing AMR in the environmental context, particularly concerning pharmaceutical manufacturing waste in India. Our findings strongly support the theoretical assumption that the effectiveness of stakeholder engagement in tackling AMR is significantly influenced by the relative power and interests of various actors within the system. Indeed, the research reveals a fragmented policy landscape, where economic constraints and capacity gaps significantly impact on industry behaviours and regulatory enforcement. These factors create a power dynamic that often favours industry interests over environmental and public health concerns. The reluctance of some government authorities to engage in the study further highlights the sensitive nature of this issue and the complex power relations at play.

Our findings indicate that bridging the gap between stakeholder perspectives is crucial for the future of AMR mitigation in the environment. The study emphasises the need for a One Health approach, which requires strong leadership and institutionalisation within governmental structures. However, the effectiveness of this approach is contingent upon balancing the diverse interests and power of stakeholders from government, industry, and civil society.

The study also highlights a critical need to enhance knowledge and awareness among policymakers and stakeholders about AMR in the environment. The power of evidence in shaping policy decisions emerged as a key theme, with stakeholders emphasising the need for robust, context-specific data to drive change.

Looking ahead, addressing AMR in the environment in India – and by extension, in other LMICs – requires a nuanced strategy that acknowledges and navigates the power dynamics among stakeholders. This includes fostering collaboration, aligning incentives, and building capacity across sectors. Future research should aim to develop strategies for effectively balancing stakeholder interests and explore how power dynamics can be harnessed to drive positive change in AMR policy and practice. In conclusion, this study not only validates the framework of stakeholder power and interests in the context of AMR but also provides valuable insights for policymakers and practitioners.

## Data Availability

This qualitative study analysed data derived from interview transcripts. These are available from the corresponding author upon reasonable request. Please note that some data may not be shared to respect participants’ privacy and their right to refuse data dissemination.

## Abbreviations

AMR: Antimicrobial Resistance
ARB: Antibiotic Resistant Bacteria
ARG: Antibiotic Resistance Gene
CDSCO: Central Drugs Standard Control Organisation
CPCB: Central Pollution Control Board
GDP: Gross Domestic Product
GMP: Good Manufacturing Practice
GOI: Government of India
IIT-B: Indian Institute of Technology Bombay
MoEFCC: Ministry of Environment Forestry and Climate Change
NAP: National Action Plan
NGO: Non-Governmental Organisation
NGT: National Green Tribunal
PNEC: Predicted No Effect Concentration
SPCB: State Pollution Control Board
UNEP: United Nations Environment Programme
VFT: Veterans Forum for Transparency in Public Life
WHO: World Health Organization
ZLD: Zero Liquid Discharge
