# Correction

**DOI:** 10.1080/16549716.2025.2502282

**Published:** 2025-05-09

**Article title: ‘Mind the gaps’: stakeholder perspectives on addressing antimicrobial resistance in the environment in the Indian context**

**Authors**: Anishka Cameron, John Connolly, Regina Esiovwa, Fiona L. Henriquez, Andrew Hursthouse, Suparna Mukherji and Soumyo Mukherji

**Journal**: *Global Health Action*

**Bibliometrics**: Volume 18, Number 1, e2491200

**DOI**: http://dx.doi.org/10.1080/16549716.2025.2491200

When this article was originally published, an incorrect URL was provided for the source of Figure 1, Yadav (2022), and it was not included in the reference list.

This has now been corrected, alongside other small changes that do not affect the academic content of the article. The publisher apologises for any errors caused.

